# Large scale text mining for deriving useful insights: A case study focused on microbiome

**DOI:** 10.3389/fphys.2022.933069

**Published:** 2022-08-31

**Authors:** Syed Ashif Jardary Al Ahmed, Nishad Bapatdhar, Bipin Pradeep Kumar, Samik Ghosh, Ayako Yachie, Sucheendra K. Palaniappan

**Affiliations:** ^1^ SBX Corporation Inc., Tokyo, Japan; ^2^ The NLP Group, The Systems Biology Institute, Tokyo, Japan

**Keywords:** microbiome, text-mining, nlp, PubMed, word2vec, hypothesis generation, disease, food

## Abstract

Text mining has been shown to be an auxiliary but key driver for modeling, data harmonization, and interpretation in bio-medicine. Scientific literature holds a wealth of information and embodies cumulative knowledge and remains the core basis on which mechanistic pathways, molecular databases, and models are built and refined. Text mining provides the necessary tools to automatically harness the potential of text. In this study, we show the potential of large-scale text mining for deriving novel insights, with a focus on the growing field of microbiome. We first collected the complete set of abstracts relevant to the microbiome from PubMed and used our text mining and intelligence platform Taxila for analysis. We drive the usefulness of text mining using two case studies. First, we analyze the geographical distribution of research and study locations for the field of microbiome by extracting geo mentions from text. Using this analysis, we were able to draw useful insights on the state of research in microbiome w. r.t geographical distributions and economic drivers. Next, to understand the relationships between diseases, microbiome, and food which are central to the field, we construct semantic relationship networks between these different concepts central to the field of microbiome. We show how such networks can be useful to derive useful insight with no prior knowledge encoded.

## 1 Introduction

Microorganisms are omnipresent and live in close association with their hosts. The microbiome is often referred to as the collection of all such microorganisms. The microbiome is increasingly understood as a primary driver and facilitator of human health (Eckburg et al. (2005)). The microbiome can be found throughout the human body, in the skin, gut, and even in the blood. The spread and diversity of the microbiota in different sites of the body have been shown to have a direct impact on overall well-being and disease states. For instance, taxonomic shifts in intestinal microbiota are shown to be strongly correlated with inflammatory bowel diseases (IBD) (Bekkers et al. (2021)).

**FIGURE 1:**
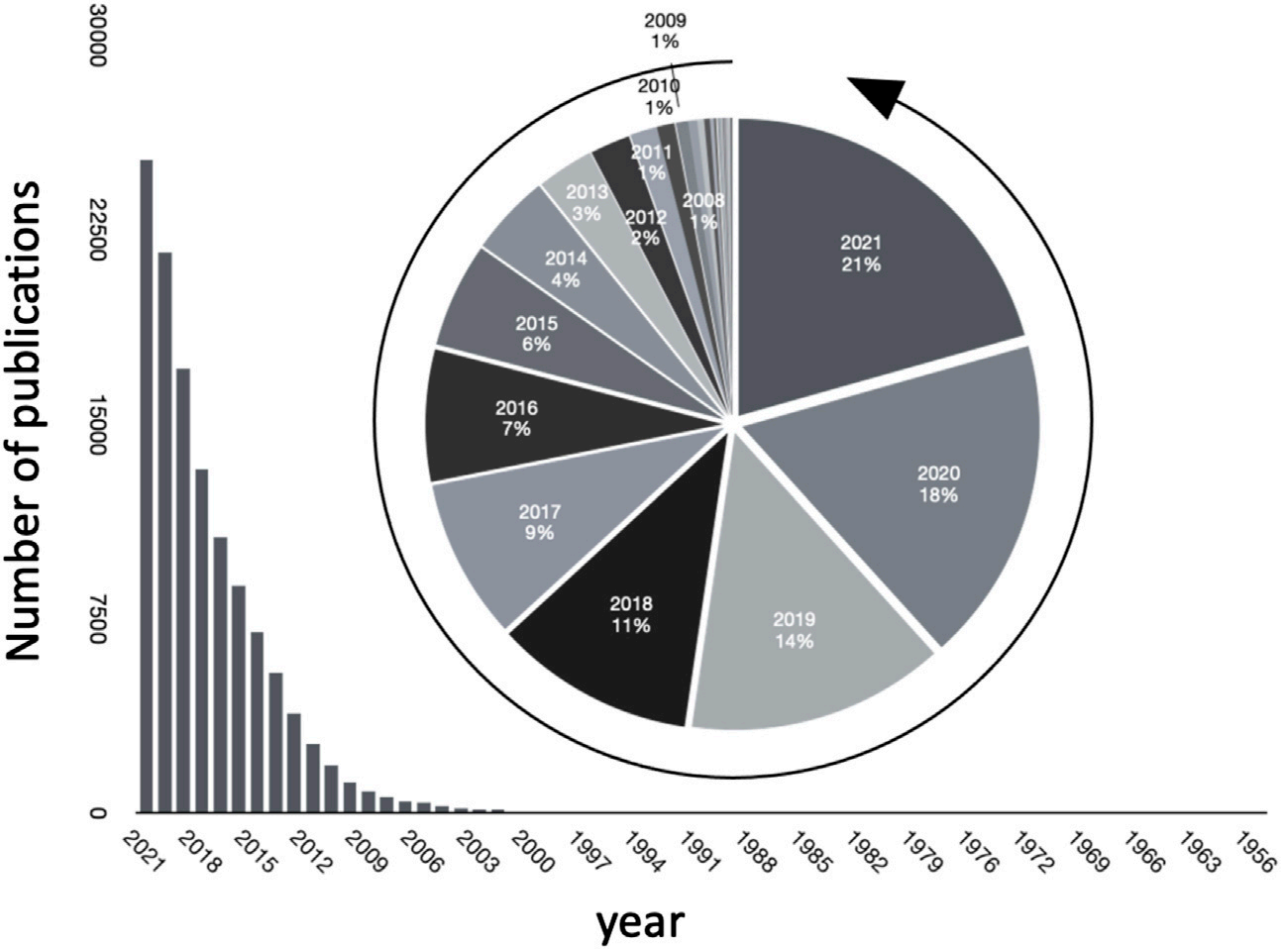
Growth of publications pertinent to microbiome in PubMed over the years.

Studying microbiomes using metagenomic sequence data through high throughput screening technologies such as 16S rRNA sequencing, whole metagenome shotgun sequencing, and their analysis is a topic of major interest and has been leading to an explosion of major discoveries. Besides this data (often termed as primary data), scientific publications store collective knowledge (referred to as secondary data) in unstructured, natural language form. The ever increasing rate of publications in the field is evident, as seen in Figure 1), where we plot the number of publications every year journaled in PubMed. Interestingly, over 20 percent of the total publications in the field have been published in 2021.

PubMed, for instance implicitly stores relationships between different concepts such as microbes, the diseases they affect, food that contribute to alterations in microbiome in the abstracts (unstructured format). Systematically extracting these relationships from text is the core thesis of the field of natural language processing (NLP) and text mining. For instance, NLP models such as Named Entity Recognizers (NERs) can identify key concepts such as Geographical location, disease terms, microorganisms name mentions etc. from text and map them onto the corresponding standard identifiers and further ascertain relationships between them. Text-mining tools in that sense help by systematically retrieve, annotate and extract existing knowledge and potentially be the basis of forming potential new hypotheses that can be used for designing future investigations.

**FIGURE 2:**
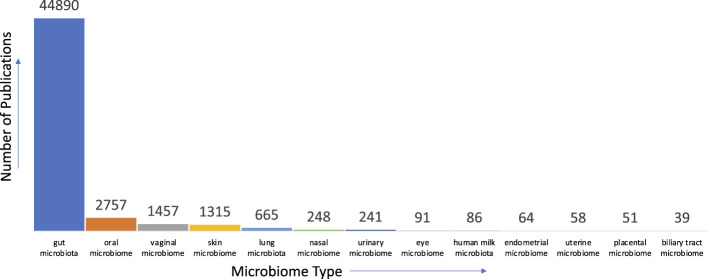
Distribution of publications pertinent to microbiome and anatomical areas in PubMed.

Given our nascent level of understanding of the microbiome, there are many avenues where text mining can aid in first gaining a succinct understanding of current knowledge, identifying gaps in them, and further aid in bridging these gaps to accelerate scientific discovery. For instance, concerning the human microbiome, there is a multitude of anatomical areas where the microbiome is prevalent and studied. When we analyzed the literature in PubMed for the distribution of studies relevant to anatomical areas, we found that the gut microbiome is the most studied (>85%) followed by oral microbiome etc (See Figure 2 for details).

With the help of two illustrative case studies, we show how text mining can be a powerful enabler. First, for our study, we focus on bibliometrics which deals with the statistical analysis of publications (Cobo et al. (2015)). Bibliometrics enables endpoints such as understanding key scientific leaders or institutions, major topics, network properties of scientific discipline among others (Sinatra et al. (2016)). Interestingly, even within the field of microbiome there have been attempts to use bibliometrics to understand publication trends (Zhu et al. (2021); Yue et al. (2020)). Our motivation stemmed from a recent study (Abdill et al. (2022)) which analyzed global repositories of human microbiome studies focused on DNA sequencing data sets and found that there was an over-representation of the developed world. Given that the analysis of such data sets often leads to scientific publications, we hypothesized that similar trends should be observed in publications too. In this paper, we perform a thorough analysis of all publication abstracts pertinent to the microbiome. Moreover, In addition to confirming existing studies (using alternate approaches than text mining), we could further draw additional insights by analyzing text given the richness of the meta-information in the publications.

For this, we analyze the mention of geographical information in text by considering text in abstracts (which we assumed is a proxy for a study focused on a geolocation) and from author affiliations (which we assume is a proxy for countries that are driving research in microbiome through research funding) to perform geographical trend analysis. Our focus was to compare funding of research vis-à-vis geo-specific studies. For this, we use custom-built named entity recognizer modules that identify geographical entities mentioned in text. Our analysis leads us to results similar to those shown in Abdill et al. (2022). Additionally, we were able to draw interesting new insights based on combining both these dimensions. More details on the methods and results are presented in further sections of the paper.

Next, interactions between key concepts relevant to microbiome, in particular, the microorganisms, the disease, and the food are being investigated extensively in numerous studies and this information is scattered in scientific literature. Manual efforts such as Li et al. (2021) and Janssens et al. (2018) focus on systematically curating such interactions from literature manually. There have been multiple studies looking at learning and identifying interactions through various text mining and supervised learning methods (Lim et al. (2016); Zafeiropoulos et al. (2022)). Co-occurrence based interaction networks have also been attempted (Freilich et al. (2010)). While most of these methods either rely on labeled data or on more traditional means of information extraction, our focus is on leveraging the recent advancements and models in the field of text mining for extracting interactions relying on unsupervised learning strategies that don’t rely on the availability of labeled training data. Specifically, we learn a shallow neural network (word2vec) which in turn learns word (phrase) embeddings (Mikolov et al. (2013)). The vectors that represent each word’s (phrase’s) embedding have implicitly learned the relationship between the words (and phrases) and hence such vectors can be compared for their relatedness using measures such as vector cosine similarity. After learning the embeddings of each word (and phrase) in the corpus, we construct word2vec embedding based networks (semantic similarity networks) based on disease, food, and microbiome terms in the corpus (obtained from running our NER models). The networks have the key concepts as node pairs with edges weighted by their cosine similarity values. A cosine threshold was used to limit the edges of the network. We show that these networks implicitly capture a lot of well-known associations between the concepts. They are useful to uncover interesting relationships between key terms and can be used for encoding prior knowledge effectively. More importantly, we also made an interactive tool available for users to explore and draw further insights.

All of the text analysis presented in this paper leverages on the text analysis and intelligence platform Taxila (Ghosh et al. (2011)). Taxila focuses on automatic collection, collation, extraction, and interpretation of knowledge from scientific communications and other diverse sources of text. It comes with powerful off-the-shelf NLP modules to harness the power of text. Additionally, practitioners and machine learning engineers in NLP can build powerful models directly into Taxila without any pre-configuration. Taxila in that sense aims to bridge the gap between domain scientists in bio-medicine and NLP researchers effectively.

In terms of the structure of the paper, Section 2 details the methodology of our approach including details of the data set we use. Section 3 applies the methodology on the microbiome data set and discusses the results we obtain. Further, Section 4 puts our results in perspective by identifying avenues of improvements and sets the stage for further research.

## 2 Methods

### 2.1 Data acquisition

For creating a text corpus, we collected PubMed abstracts. We used microbiome as the search query in PubMed to extract all abstracts into the Taxila system. Given a search query, Taxila automatically fetches from PubMed using eutils (Sayers (2009)) and stores the text data (including metadata) after preprocessing and prepares it for further processing. In total, 108,515 abstracts were extracted. Publication date ranges for the collected publications were from 18 Dec 2000 to 01 Mar 2022. The PubMed id, abstract, title, authors, and their affiliations were collected. Data was collected on 01 Mar 2022.

### 2.2 Geographical analysis of text

As discussed in previous sections, recently, it was reported that microbiome genome data sets are over-represented and focused on the developed world (specifically the United States) (Abdill et al. (2022)). While these results have spurred a lot of debate, we wanted to delve deeper to see if similar trends can be obtained purely from the analysis of publication abstracts and more importantly if further new insights can be drawn from analyzing text. Specifically, we explored the following dimensions.

• Analyze the geographical trend on the mentions of geographical information (such as cities, counties etc.) in the abstract text. We assumed that mention of geographical information in an abstract suggests a study focused on that geography.
• Analyze the geographical trend of scientific community interest in the microbiome. For this, we considered researchers who were publishing in the field of microbiome and their affiliations. Here we assume that this metric is a good proxy for research funding from the countries as well as a growing awareness of the field in the geography.
• Combine and compare analyses from one to two together to see if they led to novel insights.
• In both the above aspects we wanted to understand if there was a role of the economic status of countries. For this we used the new world bank economy classification of countries as a basis to classify countries and present our analysis (Wor (2022))

**FIGURE 3:**
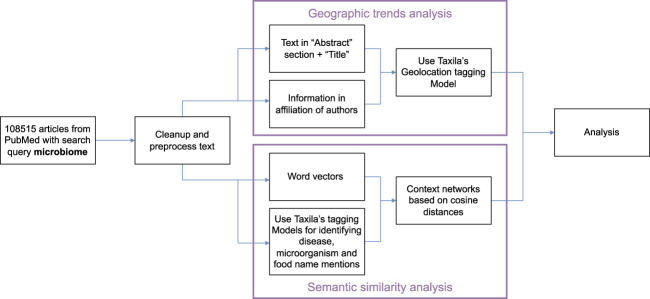
Analysis workflow for geographical trends and semantic similarity in Taxila.

Our pipeline for analysis of the above hypotheses is shown in Figure 3. Specifically, on our dataset, we first perform a round of pre-processing and clean-up of text. Next, the text analysis algorithms analyze the text in the abstract section and title of the papers and information from author affiliations. Taxila’s geolocation module analyzes these texts separately to assign a country to every article (where geolocation can be extracted). While for abstract text it is relatively straightforward to assign a geolocation i. e mention of geographical location in the abstract, for author affiliation, we consider the additional aspect that even if an author published more than one paper in the field, their name and hence their contribution to the geo statistic occurs only once. However, if an author has multiple country affiliations, then they would contribute once to all the countries they hold affiliations for. The information of the author and the affiliation is obtained from PubMed.

### 2.3 Vector based semantic similarity networks

**FIGURE 4:**
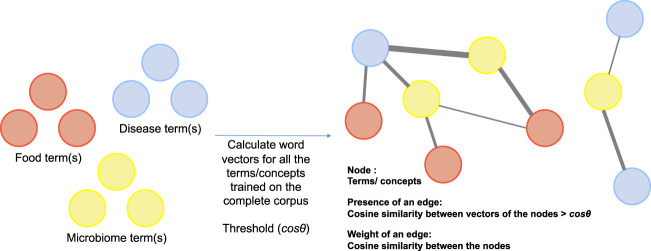
Construction of vector based semantic similarity networks.

Next, like co-occurrence networks which are common means of understanding relationship between terms (concepts) in NLP, we propose a cosine similarity network where nodes represent key terms/concepts in a corpus (in our case disease, food, and microbiome terms) and edges are a measure of the semantic similarity between the nodes. For instance, a *Cosine*_*θ*,*V*,*E*_ network would comprise of nodes represented by set *V* which are the set of key terms, an edge exists between two nodes if and only if the cosine similarity between the two nodes is above threshold cos *θ*. The set *E*, therefore, consists of all node pairs whose vector cosine similarity is more than the threshold cos *θ*. Details of the network are presented in Figure 4. This network has interesting properties and can be queried to understand the relationship between key terms in the corpus.

Our NLP pipeline for constructing these semantic similarity networks is as shown in Figure 3. Specifically, we start with a corpus of all article abstracts from PubMed for search query microbiome. Among the over 108K articles, we first perform text pre-processing and clean up. After this, we run two parallel lines of NLP tasks, one to train word2vec model on the whole corpus and another on running named entity recognizers to identify key terms pertinent to diseases, food and microbiomes (bacteria names).

For the first task, we used the vectorization module in Taxila with the following parameters to train a word2vec model. Vector length: 100, Train epoch: 10, Initial learning rate: 0.025, Window: 5, Minimum word count (in corpus): three and Algorithm: skip-gram with negative sampling (=5). For the second task, we used custom trained taggers (NERs) in Taxila for identifying disease terms, microbiome terms and food terms in the corpus.

Once these two tasks run independently, we get the vector for every term identified by the tagging module. The key terms and their vectors will now be used to create the semantic networks based on the cosine distances as described earlier.

## 3 Results

This section details the results of our analysis for both the geographical analysis and the semantic network construction analyses.

### 3.1 Geo-analysis

#### 3.1.1 United States leads the pack, followed by China and most of the developed world in microbiome research

**FIGURE 5:**
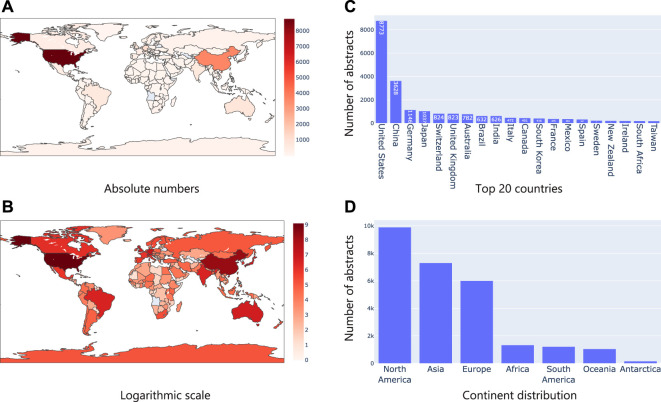
Analysis of geographical location mentions in text. **(A)** shows the number of publications in absolute numbers on a map where the intensity of the color represents the numbers; **(B)** Same statistics as **(A)**, but plotted in logarithm scale; **(C)** Top 20 country mentions; **(D)** Continent level distribution of articles.

First, we considered the text in the title and abstract of publications and identified geographical location mentions (i.e., say a city, country mention) using Taxila’s geolocation model. We then mapped them to their corresponding country. We assumed that mention of a geographical location in the abstract or title, directly refers to a microbiome study focused on the geographical area. Figure 5A shows the distribution of the articles on a geographical map. It was interesting to see that the United States and China led the pack by huge margin, followed by countries like Germany, Japan and Switzerland (top 20 countries are shown in Figure 5C). Given that the number of publications of the top two countries (United States and China) was considerably higher than the other countries, we also show Figure 5B on a logarithmic scale the distribution of countries. This image shows that even among the long tail of countries, African and South American countries (excluding Brazil) had very few microbiome studies. This led us to look at statistics w.r.t. continents. Not surprisingly, North America and Asia led the pack (riding on the United States and China respectively). As seen in the country distribution, Africa, South America, Oceania, and Antarctica had considerably less studies.

**FIGURE 6:**
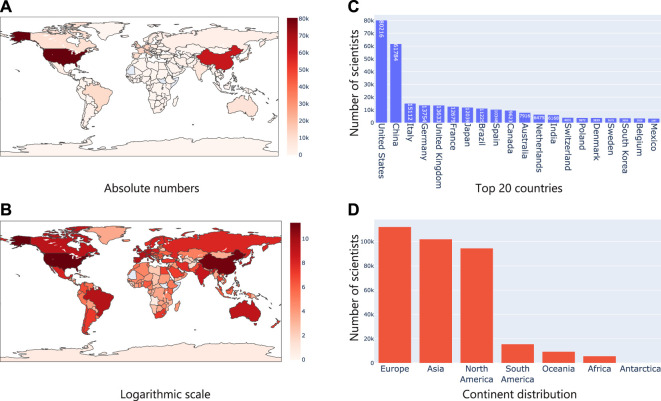
Analysis of geographical location from researcher affiliations **(A)** shows the number of authors in absolute numbers on a map where the intensity of the color represents the numbers; **(B)** Same statistics as **(A)**, but plotted in logarithm scale; **(C)** Top 20 countries based on researcher affiliations; **(D)** Continent level distribution of articles.

It was clear that the number of microbiome studies focused on specific geographies had clear differences and patterns. Next, we wanted to check if this was reflected in the geographical location derived from researchers who were working in the field of microbiome. For this, using Taxila, we extracted information about affiliation of authors publishing in microbiome and using this information, assigned a geolocation for each researcher. For instance, if a researcher is affiliated to one or more institutions in the United States, their geolocation would be the United States. Similarly, if a researcher is affiliated to institutions in multiple countries, they would be counted one time each for every country. We devised this experiment to understand if the availability of geolocation specific microbiome studies/datasets (proxy by geographical information in an abstract) correlated with corresponding increase in the number of researchers working on microbiome and subsequently to funding. As expected, we found similar trends as before. The United States and China led the pack with a considerable number of researchers active in the field of microbiome as evident in Figures 6A–C. Interestingly though, when we grouped them into continents like before, we observed that Europe led the pack followed by Asia and North America. This meant in terms of just research interest/funding (proxy by the number of researchers working in the field), Europe led the group.

**FIGURE 7:**
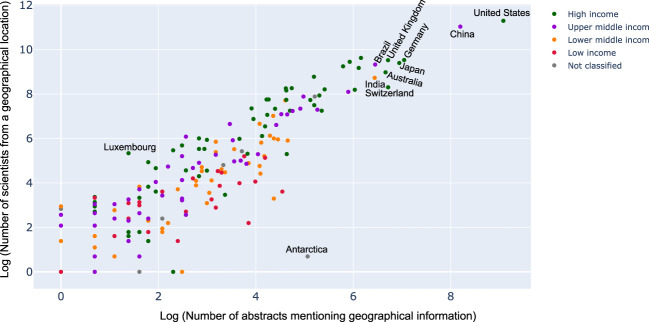
Scatter plot between the number of abstracts mentioning geographical information to the number of scientists from a geographical location (logarithmic scale). Each dot on the scatter plot describes a country and the color denotes the income classification of the country. Key countries have been labeled. For complete set of labels please refer the supplementary section.

While it seemed that countries which had geolocation mentioned in abstract and title were indeed those where a lot of research activity was observed, we wanted to see if this was always correlated. For this, we used the help of a scatter plot with the number of abstracts vs. number of scientists from a geography as shown in Figure 7. As expected, one could see a linear correlation between the two. However, it was interesting to see, for instance, Antarctica has a lot more abstracts mentioning Antarctica than the number of researchers with affiliations to it. On the other hand, countries like Luxembourg, seemed to be having a lot more researchers interested in microbiome than the amount of data sets originating from there. This was interesting and something that was not analyzed before.

#### 3.1.2 Economic stability, a key driver of microbiome research

**FIGURE 8:**
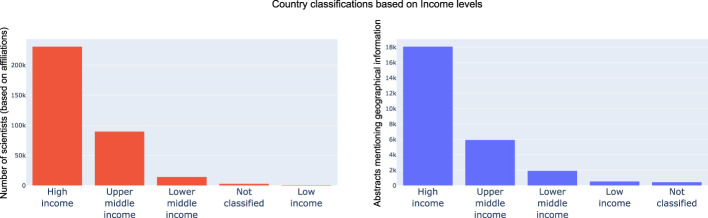
Distribution of researcher geographical profiles and geographical location mention in text grouped by economic classification of countries. As expected, High income and Upper middle income countries lead the pack by driving over 90% of research outputs and data sets.

Next, based on the outputs and analysis of the country and continent distribution, we wanted to see how much the economic stability of a country was a primary driver of research and advancements in the emerging field of microbiome. For both the dimensions explored earlier, we mapped countries into *High income*, *Upper middle income*, *Lower middle income*, *Low income* and *Not classified* based on the World Bank classification of countries (by income). In line with our hypothesis, both in-terms of microbiome data sets and researcher interests, High income countries were driving progress in the field followed by Upper middle income, Lower middle income and low-income countries. These trends are shown in Figure 8. Further we color coded countries based on their economic classification in the scatter plot, it can be observed there are clear clusters w.r.t. high income countries. It was interesting to see China, Brazil and India (from the other pack being leaders in microbiome research and output).

#### 3.2 Mining semantic associations between disease, microbiome and food

**FIGURE 9:**
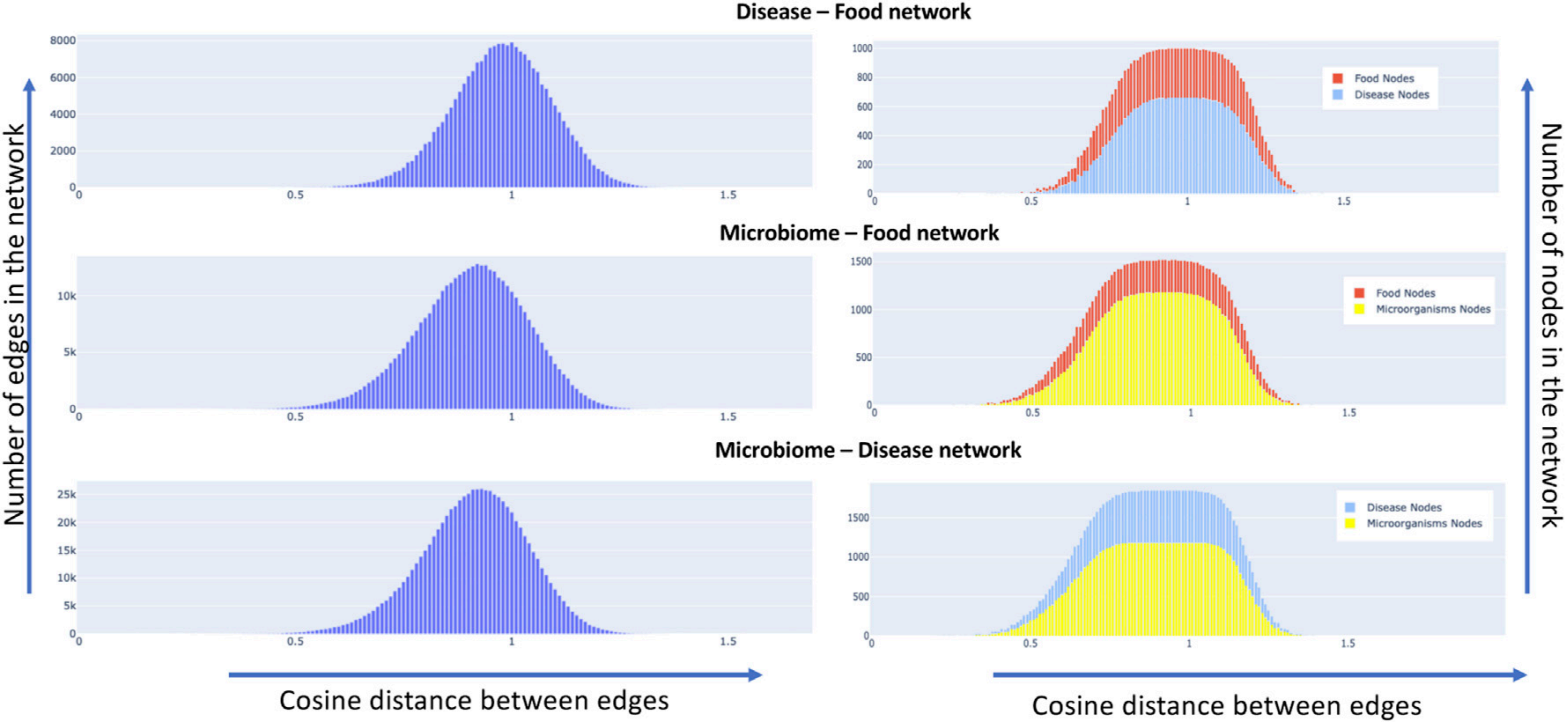
Node and edge distribution of the constructed semantic similarity networks, the left side of the plot shows for all the three concept pairs, the distribution of edges in the network as we increase the cosine distance. The right side of the graph shows the same metric w. r.t to the nodes of the network.

We constructed semantic similarity networks as described in section 2.3 for our data set. The network attributes of nodes and edges are dependent on the cosine distance threshold that is chosen. For instance, choosing a smaller value of threshold would result in fewer connected nodes and edges in the network. The nodes and edge combinations in such a network would represent a more *confident* association based on the available literature since a low value of cosine distance between two nodes indicates that they are semantically similar or related. As we increase the cosine threshold, more nodes and edge would be added to the network with a lot more false positives. To understand the effect of the increase in the threshold, we plotted the distribution of the change in edge and node characteristics. Details can be found in Figure 9, it shows the distribution of the edges (left side) and nodes (right side) of the networks as we increase the cosine distance threshold for the disease-food, microbiome-food and microbiome-disease pairs. It is interesting to note that all the network combinations show a normal distribution for edge distribution with different means. For instance, the mean of the disease-food network is closer to a cosine distance of 1, while for microbiome-disease and microbiome-food networks are much less than 1. The networks change w. r.t to the chosen cosine distance cut-off. We present a publicly available interactive portal to view these networks Syed Ashif Jardary Al Ahmed 2022a). Networks can be dynamically generated in the portal by changing the cut-off values.

It is important to note that all these networks (and relationships) have been derived in an unsupervised manner, i. e just using the text corpus only with no labeling or training data (usually needed for supervised learning models). No prior knowledge about the relationships between the microbiome, disease and food pairs were used to train the model. For us, it was interesting to see how well relationships can be learnt in this setting.

**FIGURE 10:**
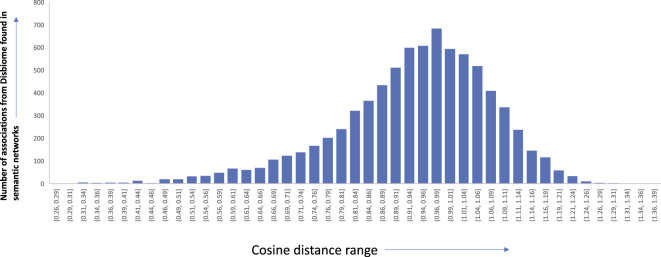
Edge distribution of the different microbiome-disease associations from Disbiome database found in our semantic networks along with the corresponding cosine distance range.

To assess the quality of the associations that were derived from the semantic networks, we set out to compare them with association networks that were manually curated from experimental data and full-text articles. We choose to compare our approach to the Disbiome database (Janssens et al. (2018)). Disbiome collects and systematically presents published microbiome-disease information. We downloaded the latest version of the database (downloaded on 7 July 2022) which consisted of microbiome-disease pairs. We wanted to check how many of these links are present in our networks, derived from just abstracts. Among the 8,456 edges in the Disbiome database, 7,910 edges were found in our semantic network (93.5% of the edges). We believe that the remaining edges are not present since the mention of the disease terms or microorganism names does not occur in the abstract of the article. Figure 10 shows the edge coverage in semantic networks with increasing cosine threshold to the associations found in Disbiome database. While it is interesting that many of the relationships were extracted only from abstracts, we believe that the accuracy can be further improved using full-text articles. Details of the edges and the cosine distances are available in the supplementary details of the paper.

**FIGURE 11:**
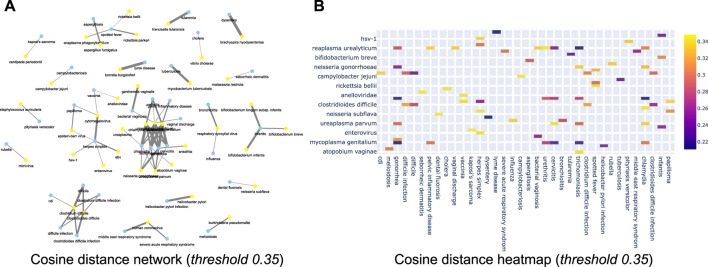
**(A)** Cosine network for cosine threshold of 0.35 of disease vs. microbiome relationship; **(B)** Cosine distance heatmap for disease vs. microbiome relationship.

Further, we now show the efficiency of the learnt relationships using a few empirical case studies. For instance, Figure 11A shows the network with a cosine threshold of 0.35 (for disease and microbiome pairs). Yellow nodes represent diseases, blue nodes represent bacterial microorganisms and an edge between a yellow node and a blue node indicates that the cosine distance between the terms is at most 0.35 i.e., they are likely to be very close to each other. For instance, in the top part of the network one can see a link between **dysentery** and ***brachyspira hyodysenteriae***. This indicates that semantically these two concepts are related to each other, as corroborated by literature evidence (Leser et al. (2000)). Similarly, Figure 11B shows the same information as a heat map where rows correspond to microorganism names and columns correspond to diseases. The intensity of the color corresponds to the cosine threshold. Similar sub-networks can be extracted for (*disease, food*), (*disease, microbiome*) and (*microbiome, food*) combinations. All of this can be used in the tool that we make public with this paper. We have also attached the network diagrams of various threshold 0.2, 0.45, 0.5, 0.6 in the supplementary section.

**FIGURE 12:**
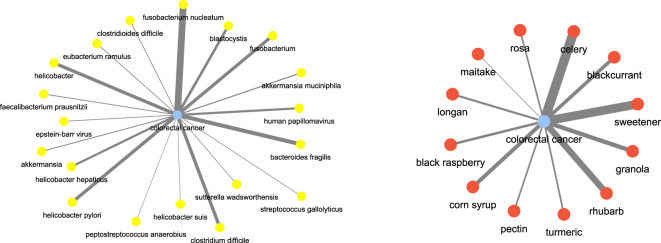
Disease and food keywords most relevant to colorectal cancer inferred from the semantic similarity network.

For instance, consider some of the (*disease, food*) and (*disease, microbiome*) combinations. We investigated **colorectal cancer** as the disease term. Figure 12 shows the disease-microbiome network. As one can see there seems to be strong link between the disease and bacterial species such as ***fusobacterium nucleatum*** (Leser et al. (2000)), ***bacteroids fragilis*** (Ulger Toprak et al. (2006)) etc which is corroborated by strong literature evidence. Similarly, for **colorectal cancer**, we wanted to check which are the food terms which had the most correlation. As it turns out, terms like **celery** (Hu et al. (1991)), **rhubarb** (Mantani et al. (2002)) and **sweetener** (Li et al. (2020)) came up as important food. The key point to take away is that these relationships have been automatically inferred only from text and no other prior knowledge was fed into the algorithm for learning.

**FIGURE 13:**
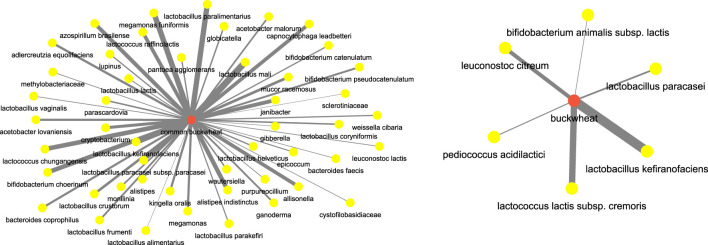
Microbiome players most correlated with buckwheat inferred from the semantic similarity network.

Next, we focused on food terms as the focus and investigated the (*food, microbiome*) aspect. For instance, to understand the microbiome participants which are correlated with **buckwheat** which has been well known for its regulatory effects on the body and gut microbiome, we investigated the network for terms **buckwheat** and **common buckwheat**. Figure 13 shows the network. We can see that bacterial species such as those of genus ***lactobacillus*** (Coman et al. (2013)), ***lactococcus*** (Shigemori et al. (2013)), ***bifidobacterium*** (Zhou et al. (2018)) are highly correlated with **buckwheat**.

**FIGURE 14:**
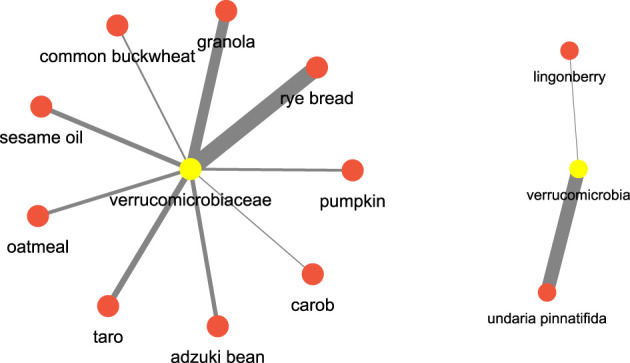
Microbiome and food keywords most relevant to verrucomicrobia inferred from the semantic similarity network.

After this let us see an example pertaining to which are the food items which have a regulatory effect on certain bacterial species. Let’s consider ***verrucomicrobia***, which are well known bacterial species known for their positive impact on the gut health, we found that food terms such as **rye bread** (Prykhodko et al. (2018)), **granola** (Ren et al. (2021)), **sesame oil** (Yuan et al. (2019)) and **seaweed** (**undaria pinntifida**) (Sichert et al. (2020)) have a high association. Figure 14 shows the network. This could imply a diet rich in these food items could have some impact on the incidence of the ***verrucomicrobia***.

While all of the illustrative case studies described above were confirmatory, we wanted to check if the networks could be used for predictive purposes. For this, we chose to focus on “Long COVID”, which is a collective term used to describe the effects and persistence of symptoms for those who have already recovered from COVID (Crook et al. (2021)). According to our semantic network, the top 10 microbiome players relevant were *human* *coronavirus*, *mycoplasma* *pneumoniae*, *actinomycetaceae*, *respiratory* *syncytial virus*, *aeromonas salmonicida*, *human adenovirus*, *sarcina ventriculi*, *arcanobacterium*, *enterovirus*, cytomegalovirus, and *corynebacterium pseudodiphtheriticum*. Next, we started looking for literature evidence implicating these microbiome players. While for most of the microbiome players, we found literature evidence, there was no explicit evidence for *actinomycetaceae*, *arcanobacterium* and *aeromonas salmonicida* in the corpus of literature that we considered for the analysis. Interestingly, we found a publication, Wang et al. (2022), which implicated bacteria of the *actinomycetaceae* in *Long* − *COVID*, it is important to note that we had only considered publications till March 2022 for this analysis. Similarly, *arcanobacterium* may be linked to the condition since they have been implicated in sepsis, pharyngitis, etc. While we do explicitly make claim of the predictive nature of these networks, we think that these networks can be the basis of devising new hypothesis generation algorithms in the future.

More importantly, we wish to highlight that we chose a limited subset of interactions to describe for the paper. Users are free to use the interactive tool, where they can interactively generate the networks by dynamically changing the threshold. The tool is hosted online and can be accessed here.

## 4 Discussions and conclusion

In this paper we show how using NLP tools, we can quickly harness large, focused text corpus to draw interesting insights with no prior knowledge. Using our text mining platform Taxila, we focus on two case studies. First, we attempted to understand the geographical distribution of microbiome research purely from information coming from abstracts (text + metadata). We were able to draw some interesting insights on the geographical distribution of microbiome research which was consistent with similar published work that focused on analyzing microbiome data sets. In terms of future work, while the focus of our analysis was purely on the frequency calculations on the microbiome subset of papers, we realize that for more meaningful insights can be drawn by normalizing the publication counts w. r.t to total papers from a country. Additionally, we did not focus our study only on human related studies, we considered the general microbiome space. Both these considerations can be the focus of future studies and currently are out of scope of this paper’s analysis.

Next, we focused on retrieving interactions and relationships between key concepts in microbiome space namely disease terms, food terms and microbes. Instead of traditional ways of extracting relationships, either through co-occurrence or training supervised machine learning models. We leveraged the method of computing word embeddings (using the word2vec algorithm) to capture the relationship between key terms and showed that the embeddings preserve relationships between these concepts. Such networks are already very useful aids for understanding the key relationships quickly and more importantly can be used as features for further focused studies (such as in Alachram et al. (2021)). We also did not compare the quality of the networks with existing hand curated manual efforts in this direction, it will interesting future work to perform a data driven comparison to assess the performance. Additionally, it would be interesting to use these embeddings to infer causality or to infer positive or negative correlations between concepts (such as in Wu et al. (2021)). Additionally, in this study we only established the quality of the learnt semantic relationships empirically since our focus was only to establish the potential of text mining algorithms to derive such relationships with no pre-training or domain understanding, a more rigorous analysis of the networks is interesting future work. Additionally, it would be interesting to compare relationships with manually curated efforts (or prior knowledge in databases) in this direction.

Additionally, linking the two analysis together i.e., geographical analysis with concepts of disease or microbiome is interesting, to study to see if there are trends on disease focus or microbiome focus.

## Data Availability

Data and code for the analyses presented in this paper are placed here. The graphs shown in the geo-analysis section can be used interactively viewed at Syed Ashif Jardary Al Ahmed (2022b). Further inquiries can be directed to the corresponding author.

## References

[B1] Abdill R. J. Adamowicz E. M. Blekhman R. (2022). Public human microbiome data are dominated by highly developed countries. PLoS Biol. 20, e3001536. 10.1371/journal.pbio.3001536 35167588 PMC8846514

[B2] Alachram H. Chereda H. Beißbarth T. Wingender E. Stegmaier P. (2021). Text mining-based word representations for biomedical data analysis and protein-protein interaction networks in machine learning tasks. PloS one 16, e0258623. 10.1371/journal.pone.0258623 34653224 PMC8519453

[B3] Bekkers M. Stojkovic B. Kaiko G. E. (2021). Mining the microbiome and microbiota-derived molecules in inflammatory bowel disease. Int. J. Mol. Sci. 22, 11243. 10.3390/ijms222011243 34681902 PMC8540913

[B4] Cobo M. J. Martínez M.-Á. Gutiérrez-Salcedo M. Fujita H. Herrera-Viedma E. (2015). 25 years at knowledge-based systems: A bibliometric analysis. Knowledge-based Syst. 80, 3–13. 10.1016/j.knosys.2014.12.035

[B5] Coman M. M. Verdenelli M. C. Cecchini C. Silvi S. Vasile A. Bahrim G. E. (2013). Effect of buckwheat flour and oat bran on growth and cell viability of the probiotic strains lactobacillus rhamnosus imc 501®, lactobacillus paracasei imc 502® and their combination synbio®, in synbiotic fermented milk. Int. J. Food Microbiol. 167, 261–268. 10.1016/j.ijfoodmicro.2013.09.015 24140807

[B6] Crook H. Raza S. Nowell J. Young M. Edison P. (2021). Long Covid—Mechanisms, risk factors, and management. bmj 374, n1648. 10.1136/bmj.n1648 34312178

[B7] Eckburg P. B. Bik E. M. Bernstein C. N. Purdom E. Dethlefsen L. Sargent M. (2005). Diversity of the human intestinal microbial flora. science 308, 1635–1638. 10.1126/science.1110591 15831718 PMC1395357

[B8] Freilich S. Kreimer A. Meilijson I. Gophna U. Sharan R. Ruppin E. (2010). The large-scale organization of the bacterial network of ecological co-occurrence interactions. Nucleic Acids Res. 38, 3857–3868. 10.1093/nar/gkq118 20194113 PMC2896517

[B9] Ghosh S. Matsuoka Y. Asai Y. Hsin K.-Y. Kitano H. (2011). Software for systems biology: From tools to integrated platforms. Nat. Rev. Genet. 12, 821–832. 10.1038/nrg3096 22048662

[B10] Hu J. Liu Y. Yu Y. Zhao T. Liu S. Wang Q. (1991). Diet and cancer of the colon and rectum: A case-control study in China. Int. J. Epidemiol. 20, 362–367. 10.1093/ije/20.2.362 1917235

[B11] Janssens Y. Nielandt J. Bronselaer A. Debunne N. Verbeke F. Wynendaele E. (2018). Disbiome database: Linking the microbiome to disease. BMC Microbiol. 18, 50–56. 10.1186/s12866-018-1197-5 29866037 PMC5987391

[B12] Leser T. D. Lindecrona R. H. Jensen T. K. Jensen B. B. Møller K. (2000). Changes in bacterial community structure in the colon of pigs fed different experimental diets and after infection with brachyspira hyodysenteriae. Appl. Environ. Microbiol. 66, 3290–3296. 10.1128/aem.66.8.3290-3296.2000 10919783 PMC92147

[B13] Li L. Jing Q. Yan S. Liu X. Sun Y. Zhu D. (2021). Amadis: A comprehensive database for association between microbiota and disease. Front. Physiol. 12, 697059. 10.3389/fphys.2021.697059 34335304 PMC8317061

[B14] Li X. Liu Y. Wang Y. Li X. Liu X. Guo M. (2020). Sucralose promotes colitis-associated colorectal cancer risk in a murine model along with changes in microbiota. Front. Oncol. 10, 710. 10.3389/fonc.2020.00710 32582527 PMC7286428

[B15] Lim K. M. K. Li C. Chng K. R. Nagarajan N. (2016). @ minter: Automated text-mining of microbial interactions. Bioinformatics 32, 2981–2987. 10.1093/bioinformatics/btw357 27312413

[B16] Mantani N. Sekiya N. Sakai S. Kogure T. Shimada Y. Terasawa K. (2002). Rhubarb use in patients treated with kampo medicines-a risk for gastric cancer? Yakugaku Zasshi 122, 403–405. 10.1248/yakushi.122.403 12087778

[B17] Mikolov T. Sutskever I. Chen K. Corrado G. S. Dean J. (2013). Distributed representations of words and phrases and their compositionality. Adv. neural Inf. Process. Syst. 26.

[B18] Prykhodko O. Sandberg J. Burleigh S. Björck I. Nilsson A. Fåk Hållenius F. (2018). Impact of rye kernel-based evening meal on microbiota composition of young healthy lean volunteers with an emphasis on their hormonal and appetite regulations, and blood levels of brain-derived neurotrophic factor. Front. Nutr. 5, 45. 10.3389/fnut.2018.00045 29896479 PMC5986961

[B19] Ren G. Fan X. Teng C. Li Y. Everaert N. Blecker C. (2021). The beneficial effect of coarse cereals on chronic diseases through regulating gut microbiota. Foods 10, 2891. 10.3390/foods10112891 34829172 PMC8620804

[B20] Sayers E. (2009). The e-utilities in-depth: Parameters, syntax and more. Entrez Programming Utilities Help. *[Internet]* .

[B21] Shigemori S. Yonekura S. Sato T. Otani H. Shimosato T. (2013). Expression of the immunoreactive buckwheat major allergenic storage protein in lactococcus lactis. Appl. Microbiol. Biotechnol. 97, 3603–3611. 10.1007/s00253-012-4608-9 23212674

[B22] Sichert A. Corzett C. H. Schechter M. S. Unfried F. Markert S. Becher D. (2020). Verrucomicrobia use hundreds of enzymes to digest the algal polysaccharide fucoidan. Nat. Microbiol. 5, 1026–1039. 10.1038/s41564-020-0720-2 32451471

[B23] Sinatra R. Wang D. Deville P. Song C. Barabási A.-L. (2016). Quantifying the evolution of individual scientific impact. Science 354, aaf5239. 10.1126/science.aaf5239 27811240

[B24] Syed Ashif Jardary Al Ahmed S. K. P. (2022a). Interactive visualizer of semantic networks for microbiome. Availableat: http://distances.mbpaper.taxila.io/ (Accessed June 30th, 2022).

[B25] Syed Ashif Jardary Al Ahmed S. K. P. (2022b). Visualization for bibiliometrics of microbiome literature. Availableat: http://charts.mbpaper.taxila.io/ (Accessed June 30th, 2022).

[B26] Ulger Toprak N. Yagci A. Gulluoglu B. Akin M. Demirkalem P. Celenk T. (2006). A possible role of bacteroides fragilis enterotoxin in the aetiology of colorectal cancer. Clin. Microbiol. Infect. 12, 782–786. 10.1111/j.1469-0691.2006.01494.x 16842574

[B27] Wang B. Zhang L. Wang Y. Dai T. Qin Z. Zhou F. (2022). Alterations in microbiota of patients with Covid-19: Potential mechanisms and therapeutic interventions. Signal Transduct. Target. Ther. 7, 143. 10.1038/s41392-022-00986-0 35487886 PMC9052735

[B28] Wor (2022). World bank country classifications by income level (Accessed 30 04, 2022).

[B29] Wu C. Xiao X. Yang C. Chen J. Yi J. Qiu Y. (2021). Mining microbe–disease interactions from literature via a transfer learning model. BMC Bioinforma. 22, 432. 10.1186/s12859-021-04346-7 PMC8430297 34507528

[B30] Yuan T. Chu C. Shi R. Cui T. Zhang X. Zhao Y. (2019). Apoe-dependent protective effects of sesamol on high-fat diet-induced behavioral disorders: Regulation of the microbiome-gut–brain axis. J. Agric. Food Chem. 67, 6190–6201. 10.1021/acs.jafc.9b01436 31117496

[B31] Yue Y.-Y. Fan X.-Y. Zhang Q. Lu Y.-P. Wu S. Wang S. (2020). Bibliometric analysis of subject trends and knowledge structures of gut microbiota. World J. Clin. Cases 8, 2817–2832. 10.12998/wjcc.v8.i13.2817 32742991 PMC7360702

[B32] Zafeiropoulos H. Paragkamian S. Ninidakis S. Pavlopoulos G. A. Jensen L. J. Pafilis E. (2022). Prego: A literature and data-mining resource to associate microorganisms, biological processes, and environment types. Microorganisms 10, 293. 10.3390/microorganisms10020293 35208748 PMC8879827

[B33] Zhou X.-L. Yan B.-B. Xiao Y. Zhou Y.-M. Liu T.-Y. (2018). Tartary buckwheat protein prevented dyslipidemia in high-fat diet-fed mice associated with gut microbiota changes. Food Chem. Toxicol. 119, 296–301. 10.1016/j.fct.2018.02.052 29481895

[B34] Zhu X. Hu J. Deng S. Tan Y. Qiu C. Zhang M. (2021). Bibliometric and visual analysis of research on the links between the gut microbiota and depression from 1999 to 2019. Front. Psychiatry 11, 587670. 10.3389/fpsyt.2020.587670 33488420 PMC7819979

