# Supplementary figures and images for: Large scale text mining for deriving useful insights: A case study focused on microbiome
Source: Front Physiol. 2022 Aug 31;13:933069. doi: 10.3389/fphys.2022.933069 (PMC9473635; doi:10.3389/fphys.2022.933069)

## Disease- Microbiome Network

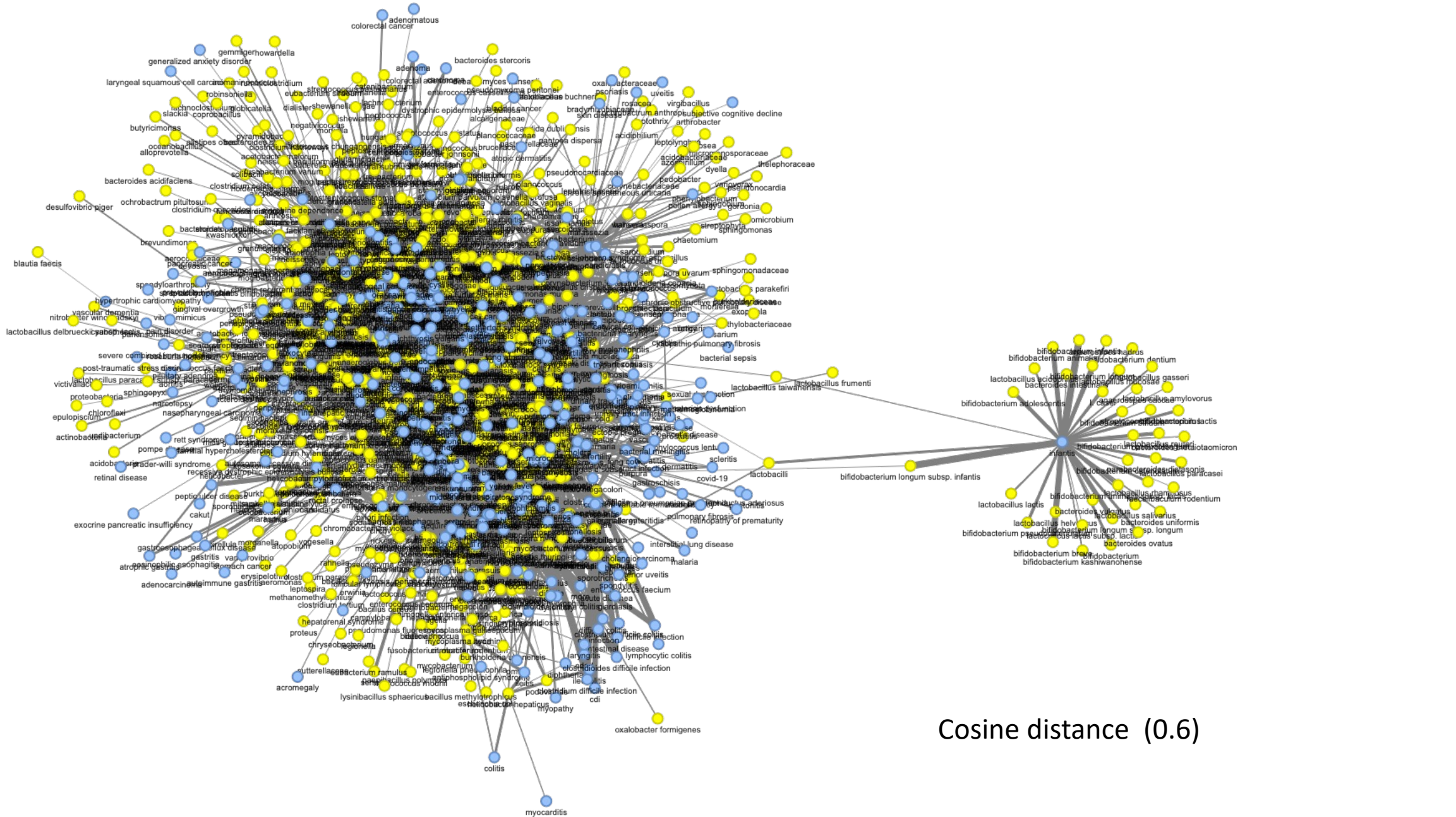

Supplement: Supplementary file 1 [file Image2.pdf]

# Disease- Microbiome Network

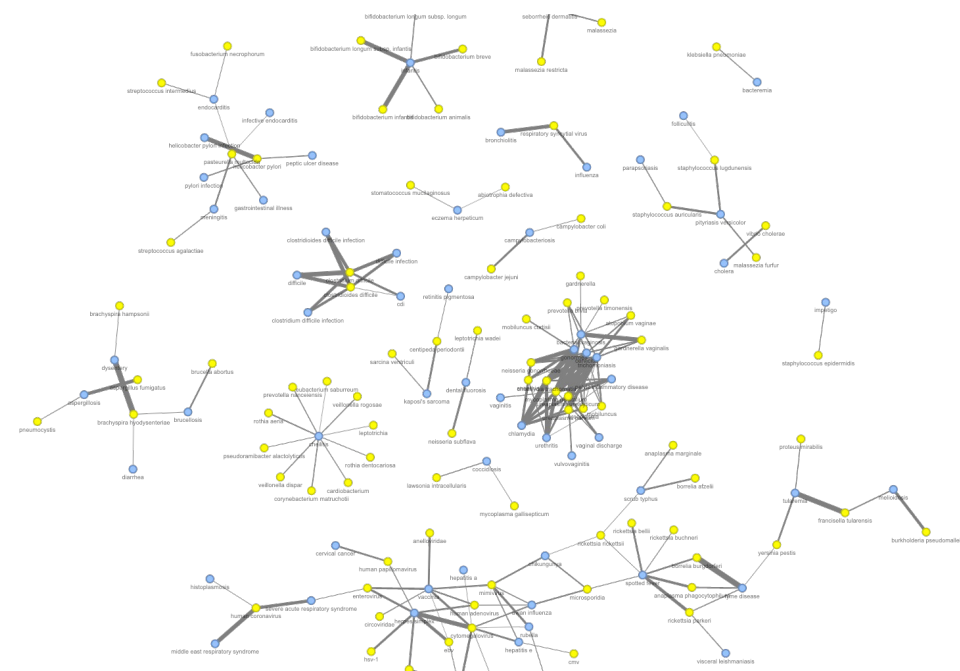

Cosine distance (0.4)

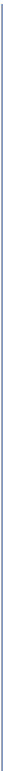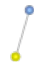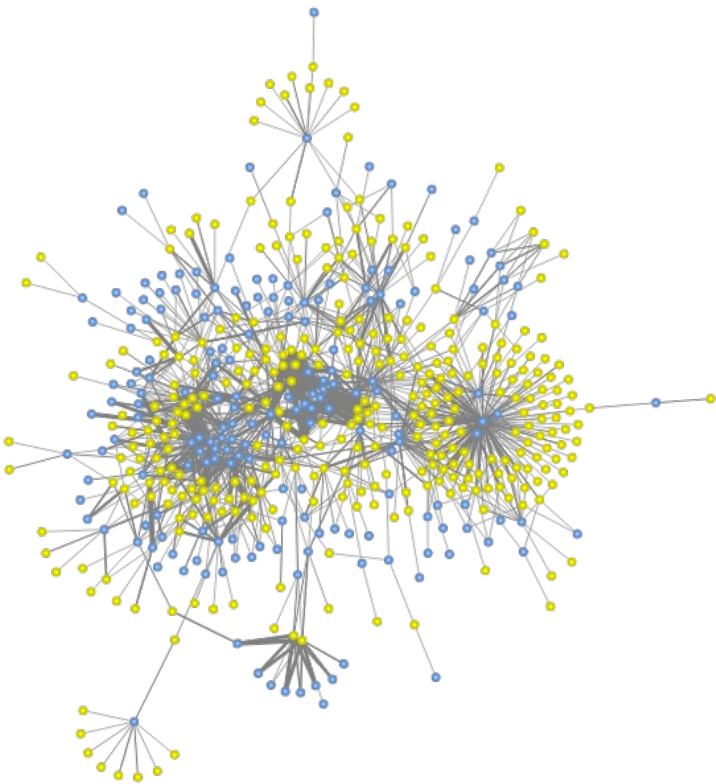

Cosine distance (0.5)

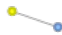

Supplement: Supplementary file 3 [file Image1.pdf]
